# The etomidate analog ET-26 HCl retains superior myocardial performance: Comparisons with etomidate in vivo and in vitro

**DOI:** 10.1371/journal.pone.0190994

**Published:** 2018-01-11

**Authors:** Xingxing Liu, Haibo Song, Jun Yang, Cheng Zhou, Yi Kang, Linghui Yang, Jin Liu, Wensheng Zhang

**Affiliations:** 1 Department of Anesthesiology, Affiliated Hospital of Zunyi Medical University, Zunyi, Guizhou, P.R. China; 2 Laboratory of Anesthesia & Critical Care Medicine, Translational Neuroscience Center, West China Hospital, Sichuan University, Chengdu, Sichuan, P.R. China; 3 Department of Anesthesiology, West China Hospital, Sichuan University, Chengdu, Sichuan, P.R. China; Indiana University School of Medicine, UNITED STATES

## Abstract

**Objective:**

(*R*)-2-methoxyethyl1-(1-phenylethyl)-1H-imidazole-5-carboxylate hydrochloride (ET-26 HCl) is a novel etomidate analogue. The purpose of this study was to characterize whether ET-26 HCl could retain the superior myocardial performance of etomidate *in vivo* and *in vitro*.

**Methods:**

*In vivo*, the influence of ET-26 HCl and etomidate on the cardiac function of dogs was confirmed using echocardiography and electrocardiogram. *In vitro*, a Langendorff preparation was used to examine direct myocardial performance in isolated rat hearts, and a whole-cell patch-clamp technique was used to study effects on the human ether-a-go-go-related gene (hERG) channel.

**Results:**

*In vivo*, after a single bolus administration of ET-26 HCl or etomidate, no significant difference in echocardiography and electrocardiogram parameters was observed. No arrhythmia occurred and no QT interval prolongation happened during the study period. In the *in vitro* Langendorff preparation, none of the cardiac parameters were abnormal, and the hERG recordings showed that ET-26 HCl and etomidate inhibited the tail current of the hERG in a concentration-dependent manner with an IC_50_ of 742.51 μM and 263.60 μM, respectively.

**Conclusions:**

In conclusion, through an *in vivo* experiment and a whole organ preparation, the current study found that ET-26 HCl can maintain a myocardial performance that is similar to that of etomidate. In addition, the electrophysiology study indicated that ET-26 HCl and etomidate inhibited the hERG at a supra-therapeutic concentration.

## Introduction

**Fig 1:**
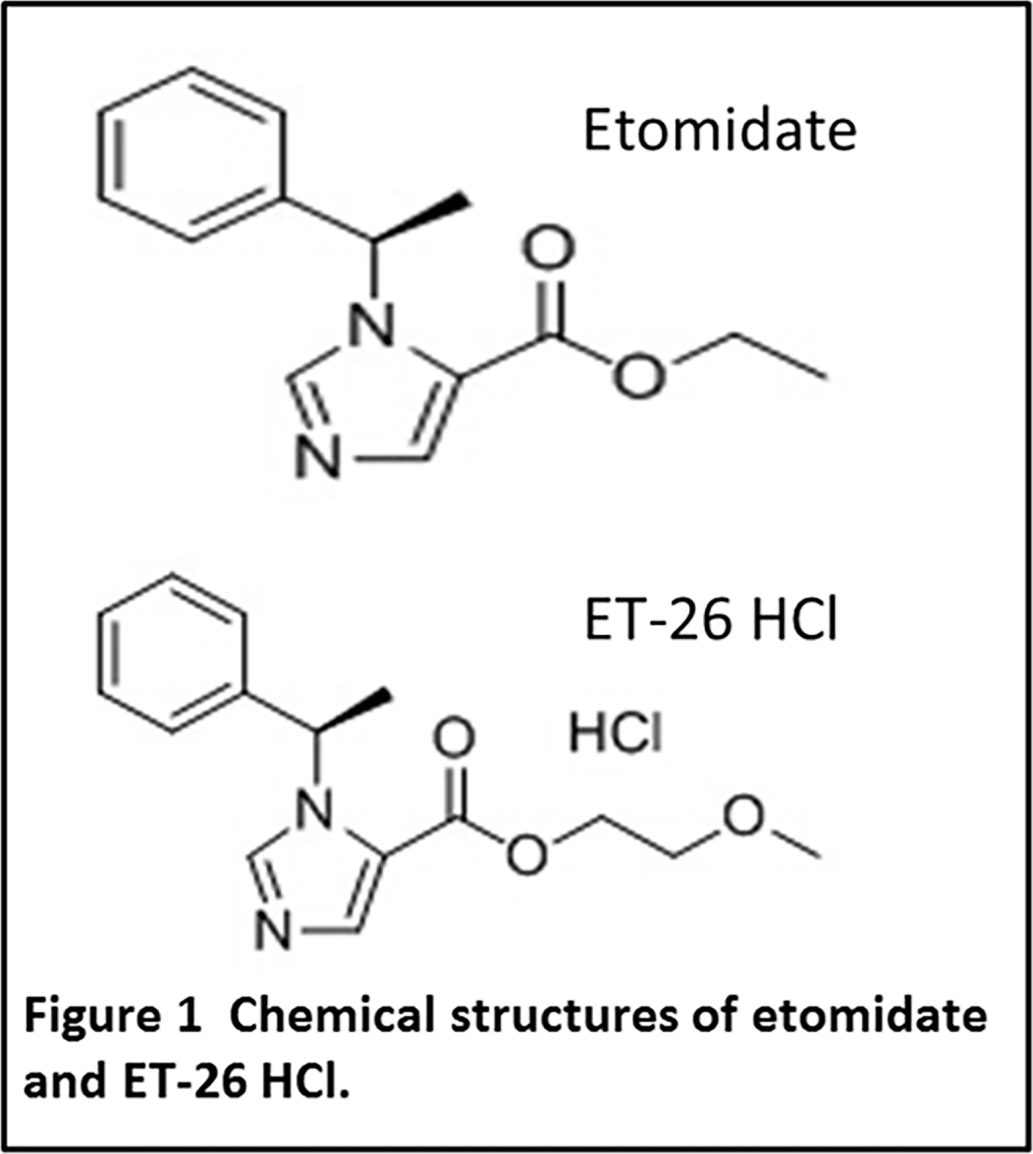
Chemical structures of etomidate and ET-26 HCl.

Etomidate [R-1-(1-ethylphenyl) imidazole-5-ethyl ester] (Fig 1) is an imidazole-based intravenous hypnotic agent. It was synthesized by Janssen Pharmaceuticals (a division of Ortho-McNeil-Janssen Pharmaceuticals, Titusville, New Jersey, USA) in 1965 [1]and particularly because of its favourable myocardial performance, it emerged in the clinical literature and gained popularity soon afterward. Unfortunately, etomidate remains controversial because it inhibits the activity of adrenal mitochondrial 11-β-hydroxylase, inducing adrenal suppression, which leads to limitation of its clinical application [2–6].

In recent years, researchers from Massachusetts General Hospital (Boston, Massachusetts, USA) have developed a series of etomidate analogues, from methoxycarbonyl-etomidate, carboetomidate, and methoxycarbonyl-carboetomidate to cyclopropyl-methoxycarbonyl metomidate, which are designed not to suppress adrenocortical function [7–10].Our laboratory has also been working on developing rational and optimal analogues of etomidate based on the hypothesis that the analogues should retain the superior myocardial performance of etomidate, but alleviate its adrenocortical suppression. Among these analogues, (*R*)-2-methoxyethyl1-(1-phenylethyl)-1H-imidazole-5-carboxylate hydrochloride (ET-26 HCl) (Fig 1) has been shown in our previous studies to possess many outstanding characteristics and to exert reduced corticosteroid suppression [11–15]. However, few studies have investigated the effects of etomidate analogues on myocardial performance, including echocardiography, ventricular repolarization, proarrhythmic risk and electrophysiology assessments.

When QT (time from the beginning of the QRS complex to the end of the T wave of the electrocardiogram, which represents the duration of the depolarization and repolarization of the ventricles) prolongation occurs, there is an increased risk of lethal ventricular tachyarrhythmia known as “torsades de pointes (TdP)” [16]. In 2005, the International Conference on Harmonization of Technical Requirements for Registration of Pharmaceuticals for Human Use issued a guideline, S7B, which recommends that new chemical entities should be assessed for the potential of delayed ventricular repolarization in animal models [17]. In recent years, in drug development, more emphasis has been placed on the potential arrhythmias that are associated with QT interval prolongation. It is generally accepted that the majority of compounds that prolong the QT interval block the cardiac rapid delayed rectifier potassium current (I_Kr_), encoded by the human-ether-a-go-go-related gene (hERG), which plays a critical role in defining ventricular repolarization [18–19].

We hypothesized that ET-26 HCl may retain the superior myocardial performance of etomidate, and in the present study, we evaluated the effect of ET-26 HCl on myocardial performance *in vivo* and *in vitro*.

## Materials and methods

### Ethics statement

All animal studies were conducted with the approval of the Committee of Scientific Research and the Institutional Animal Experimental Ethics Committee of the West China Hospital (Sichuan University, Chengdu, China) and all ethical approval decisions were based on the recommendations in their guidelines (publication number 2015015A).

### Animals

Adult Sprague-Dawley rats weighing 250–350 g and Beagle dogs weighting 8–10 kg were purchased from DOSSY Experimental Animals Co. Ltd. (Sichuan, China). They were housed with a 12/12 h light cycle, a controlled temperature of 21 ± 5°C, and a relative humidity of 55 ± 15% in the Animal Experimental Centre of the West China Hospital of Sichuan University (Sichuan, China). At the conclusion of these experiments, dogs were euthanized with an overdose of sodium pentobarbital (i.v; 100 mg/kg).

### Sources and formulation of hypnotic drugs

(*R*)-2-methoxyethyl1-(1-phenylethyl)-1H-imidazole-5-carboxylate hydrochloride (ET-26 HCl) (*Publication Patent Number*: *CN103739553 B*) was synthesized (> 99% purity) in our laboratory, as previously described [11]. Etomidate (Etomidate Lipuro^®^, B.Braun, 2 mg/mL) was purchased from Melsungen AG.

### *In vivo* studies

In our previous experiment, the anesthetic potencies of ET-26 HCl (ED_50_ = 1.44 mg/kg) and etomidate (ED_50_ = 0.43 mg/kg) were determined using the up and down method.^11^

#### *In vivo* cardiac function in dogs

**Fig 2:**
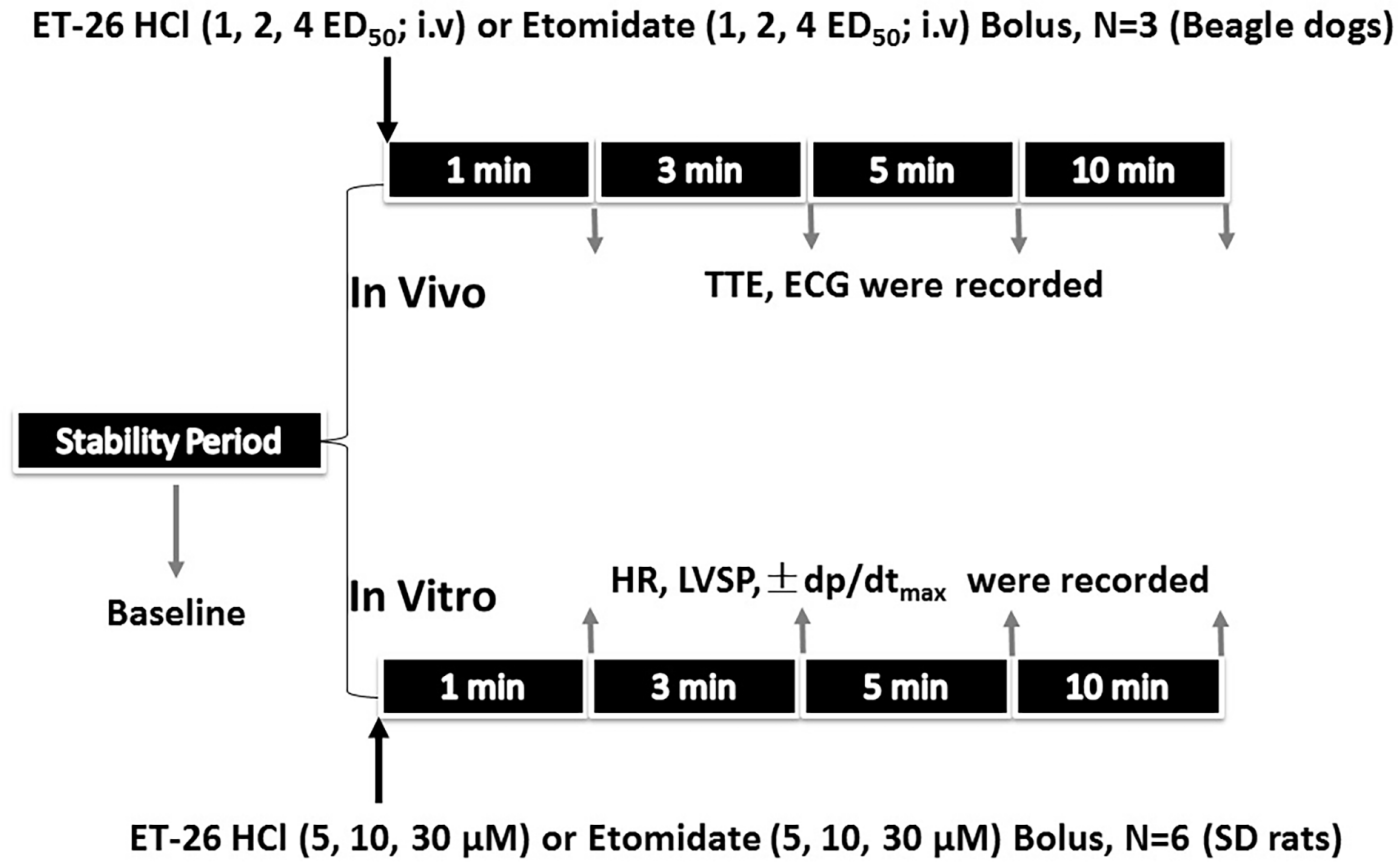
Schematic diagrams depicting the *in vivo* and *in vitro* cardiac function experimental protocol. ED_50_: 50% effective dose; TTE: echocardiography; ECG: electrocardiogram; HR: heart rate; LVSP: left ventricular systolic pressure; ± dP/dt_max_: maximal rate for left ventricular pressure rising and declining. SD: Sprague-Dawley.

For the *in vivo* experiments, a total of 18 (9 male and 9 female) Beagle dogs, were randomly assigned to ET-26 HCl treatment (ET-26 HCl group; 1×, 2×, 4× ED_50_ i.v; n = 3) or etomidate treatment (etomidate group; 1×, 2×, 4× ED_50_ i.v; n = 3). The influence of the treatments on cardiac function was confirmed using echocardiography and an electrocardiogram. After baseline, the cardiac parameters were recorded at 1, 3, 5 and 10 min following the administration of ET-26 HCl or etomidate. The detailed experimental protocol is shown in Fig 2.

#### Echocardiography

Transthoracic echocardiography (TTE) was continuously performed during the *in vivo* experiment. Beagle dogs were placed in the left lateral decubitus position under light sedation through inhalation of the lowest possible dose of isoflurane (initially 5%, then 2% to 3%) mixed with oxygen. Images were acquired using a 2–4 MHz transducer connected to the Mindray echocardiography machine (TTE, M7 series, Mindray Bio-medical Electronics Co, Ltd., Shenzhen, China). For continuous recording and to reduce experimental variability, the transducer was fixed using an angle-adjustable pedestal (*Publication Patent Number*: *CN102824192 B*), which can be pasted and prevents transducer dislodgement.

For the recording of the left ventricle (LV) parameters, the LV internal diameter in diastole and systole (LVIDd and LVIDs) was measured using M-mode echocardiography at the level of papillary muscle, following the recommendations of Bonagurra [20]. End-diastolic/systolic volume (EDV/ESV) was calculated using the Teicholz formula [21]: V = 7D^3^/ (2.4 + D), with D = LVIDd or LVIDs, respectively. LV ejection fraction (EF) was calculated as EDV − ESV/EDV.

#### Electrocardiography

A lead II electrocardiogram (ECG) was recorded using electrocardiography (MAC3500, General Care International, Shanghai, China) continuously in the *in vivo* experiment. The measurements focused on the PR interval, QRS complex, QT interval (time from the beginning of the QRS complex to the end of the T wave), QTcB (Bazett’s heart rate corrected QT interval [22], QTcB = QT/(HR)^½^) and the incidence of pharmaceutical-induced arrhythmia. The judgment of the presence of arrhythmia was in accordance with the Lambeth Conventions and Lambeth Conventions (II), and the quantification of the arrhythmias used the modified scoring system of Cuertis and Walker (Table 1)[23–24].

**Table 1:** The arrhythmia scoring system.

Arrhythmia Scores	Type of Arrhythmia
**0**	No arrhythmia
**1**	occasional (1–2 episodes within 60 s) PVC
**2**	Frequent (≥3 episodes within 60 s) PVC
**3**	1–2 episodes of VT
**4**	≥3 episodes of VT or occasional VF
**5**	Frequent VF or death

PVC: premature ventricular contraction; VT: ventricular tachycardia; VF: ventricular fibrillation.

### *In vitro* studies

#### Langendorff preparation with rat hearts

A Langendorff heart preparation (ML176-220, AD instruments, Ltd, Shanghai, China) was used to evaluate the effects of ET-26 HCl and etomidate on cardiac performance. A total of 36 (18 male and 18 female rats) isolated rat hearts were randomly assigned to an ET-26 HCl group (n = 6 for each concentration of 5, 10 and 30 μM) or etomidate group (n = 6 for each concentration of 5, 10 and 30 μM). After anesthetising the rats using sodium pentobarbital (40 mg/kg; i.p.), the rat hearts were quickly removed and mounted on a Langendorff perfusion system via the aorta at a constant pressure of 70 mmHg using Krebs-Henseleit buffer containing (in mM): NaCl 118.0, NaHCO_3_ 25.0, KCl 4.7, KH_2_PO_4_ 1.2, CaCl_2_ 2.5, MgSO_4_ 1.2, glucose 11.0, EDTA-Na_2_ 0.125 and the pH was maintained at 7.40 ± 0.05. The solution was continuously oxygenated using a mixture of oxygen (95%) and carbon dioxide (5%) in a 37°C water bath (LE 13026 thermostat, Harvard Apparatus, Ltd, Shanghai, China).

After a 30-min stabilization period, ET-26 HCl or etomidate was administered into the inflow perfusate at a concentration of 5, 10 or 30 μM. Cardiac parameters, as follows: (1) heart rate (HR); (2) left ventricular systolic pressure (LVSP), and (3) maximal rate for left ventricular pressure rise and decline (± dP/dt_max_), were measured before treatment (baseline) and at 1, 3, 5, and 10 min after hypnotic treatment. The experimental protocol is shown in Fig 2.

#### hERG recordings

The *in vitro* I_Kr_ assay, using a whole-cell patch-clamp technique [25], was designed to examine the effects of ET-26 HCl and etomidate on the hERG ion channel currents. Whole-cell recordings were obtained at room temperature using 3 to 5 MΩ patch pipettes and an Axopatch 200B amplifier in a HEPES-buffered bath solution composed of 145 mM NaCl, 4 mM KCl, 1 mM MgCl_2_, 2 mM CaCl_2_, 10 mM HEPES, and 10 mM glucose, pH 7.4. An internal solution contained 140 mM KCl, 1 mM MgCl_2_, 10 mM HEPES, 5 mM EGTA, 4 mM Na_2_-ATP, pH 7.2.

Whole-cell hERG currents expressed in human embryonic kidney (HEK-293) cells were elicited by a 2 s depolarizing pulse to +50 mV from a holding potential of −80 mV and repolarization to −50 mV for 3 s to measure the tail currents in an interval of 30 s. In the absence and presence of ET-26 HCl and etomidate, their effect on the hERG tail current was recorded. Based on a preliminary experiment, the used concentrations were 30, 100, 300, 1000, and 3000 μM for ET-26 HCl and 10, 30, 100, 300, and 1000 μM for etomidate. The inhibition percentage (%) of the hERG tail current was calculated using the following equation: inhibition percentage (%) = {1 − (remaining current amplitude)/ (control current amplitude)} × 100. The results are presented as mean ± standard deviation (SD). The IC_50_, the concentration at which a compound inhibits 50% of IKr activity, was fitted using a logistic equation.

## Statistical analysis

Data concerning the cardiac parameters *in vivo* are presented as mean ± SD. The differences among groups were compared using independent-samples t-tests. Repeated-measures ANOVA was performed to analyse the changes in several variables over time, such as LVEF, LV internal dimension and end-diastolic/systolic volume. For the *in vitro* Langendorff preparation, data are presented as mean ± SD relative to baseline values (percentage) and analysed using independent-samples t-tests. *P* < 0.05 is considered statistically significant. The statistical analysis was performed using the Statistical Package for Social Sciences (SPSS™), Windows version 16.0 (SPSS Inc, Chicago, IL).

## Results

### *In vivo* studies

#### *In vivo* cardiac function in dogs

For the *in vivo* hemodynamic study, a total of 18 Beagle dogs, with a mean weight of 9 kg (range 7–10.5 kg) and age of 8–10 months, were used to evaluate the influence of ET-26 HCl and etomidate on cardiac function using echocardiography and ECG. Table 2 shows the basal cardiac parameters before treatment.

**Table 2:** General and *in vivo* baseline cardiac parameters.

Variables	etomidate (n = 9)	ET-26 HCl (n = 9)
**Body weight, kg**	8.9 ± 1.4	9.3 ± 0.7
***TTE parameters***		
**LVIDd, mm**	32.8 ± 2.3	33.8 ± 3.2
**EDV, ml**	36.0 ± 8.1	39.7 ± 11.9
**LVIDs, mm**	23.0 ± 3.4	23.2 ± 3.5
**ESV, ml**	12.9 ± 5.7	13.3 ± 6.1
**EF, %**	65.0 ± 9.8	67.6 ± 6.9
***ECG parameters***		
**HR, bpm**	164 ± 9.8	159 ± 12.6
**PR, ms**	86 ± 4.4	85 ± 5.1
**QRS, ms**	35 ± 4.1	37 ± 4.0
**QT, ms**	246 ± 14.6	233 ± 17.4
**QTc, ms**	404 ± 20.4	378 ± 28.2

Data are presented as mean ± standard deviation. LVIDd and LVIDs: LV internal diameter in diastole and systole; EDV/ESV: End-diastolic/ systolic volume; EF: Ejection fraction; HR: heart rate; PR: PR interval; QRS: QRS complex; QT: QT interval; QTc: heart rate corrected QT interval. Nine beagle dogs were studied in each group.

**Fig 3:**
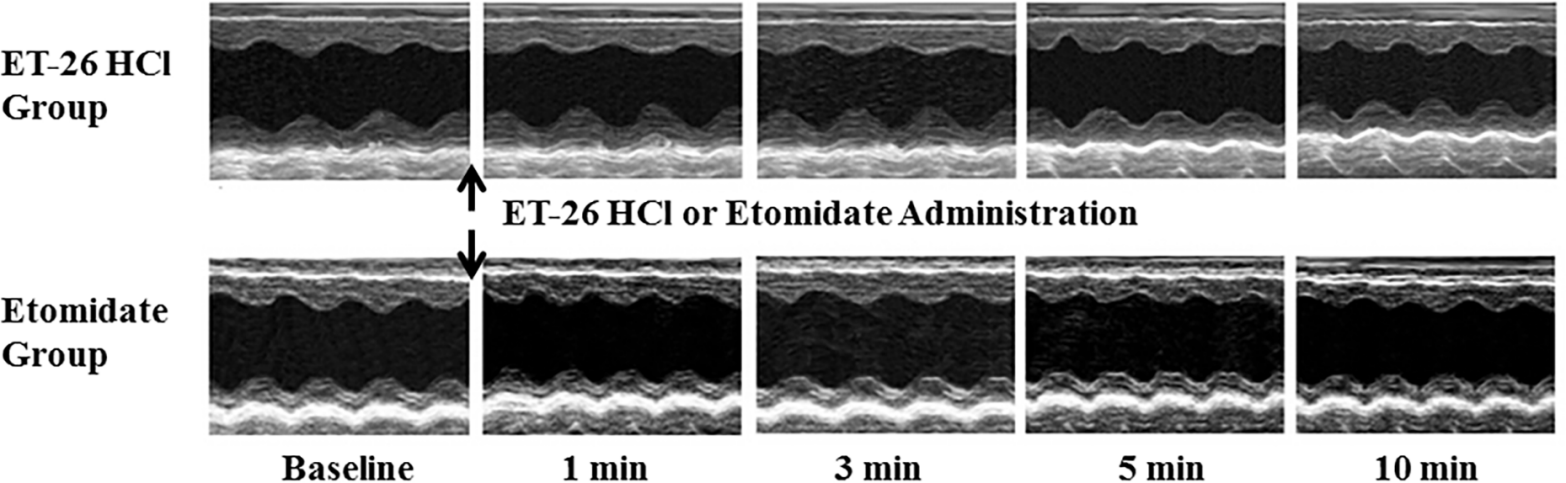
Representative M-mode echocardiogram tracing of Beagle dogs treated using 2× ED_50_ of ET-26 HCl and etomidate. Baseline = before treatment; 1, 3, 5, and 10 min represent the time after ET-26 HCl or etomidate administration. After treatment, echocardiographic parameters are similar to the pre-dose recording in both groups.

After treatment using different doses of ET-26 HCl and etomidate, no significant change was found in TTE parameters (Table 3). Fig 3 displays a representative M-mode echocardiogram tracing of Beagle dogs treated using 2× ED_50_ of ET-26 HCl and etomidate, showing that the echocardiographic parameters at 1, 3, 5, and 10 min after hypnotic administration are similar to the pre-dose recording. Table 4 shows that none of the ECG parameters during the observing time revealed any abnormal results. No arrhythmia occurred and no prolongation of QT interval prolongation was observed (Table 5).

**Table 3:** TTE parameters before and after bolus administration of etomidate and ET-26 HCl.

Group	LVIDd (mm)		EDV (ml)		LVIDs (mm)		ESV (ml)		EF (%)	
	Before	After	Before	After	Before	After	Before	After	Before	After
***Etomidate***										
**1 ED**_**50**_	33±3.9	34±4.6	37±13.7	41±17.1	24±5.1	24±6.8	14±8.8	16±11.5	63±13.0	63±18.2
**2 ED**_**50**_	33±2.3	33±2.7	37±7.8	36±8.9	23±3.2	23±4.1	12±5.3	13±7.0	67±9.0	65±11.0
**4 ED**_**50**_	32±0.8	33±1.0	34±2.5	37±3.5	23±3.0	24±2.6	12±4.6	13±4.3	65±11.8	63±11.0
***ET-26 HCl***										
**1 ED**_**50**_	34±2.5	34±3.2	39±8.5	41±11.9	23±3.4	24±3.6	14±5.4	15±6.3	69±6.0	64±8.1
**2 ED**_**50**_	33±3.2	32±2.6	36±10.9	36±8.1	21±2.4	21±2.2	10±3.6	10±3.0	71±2.0	73±1.9
**4 ED**_**50**_	32±0.9	30±2.4	34±2.6	28±7.0	22±2.6	22±3.3	12±4.1	11±5.1	65±9.6	63±8.3

Data are presented as mean ± standard deviation. ED_50_: median effective dose. TTE: echocardiography; LVIDd and LVIDs: LV internal diameter in diastole and systole; EDV/ESV: End-diastolic/systolic volume; EF: Ejection fraction. n = 3 for each sub-group.

**Table 4:** ECG parameters before and after bolus administration.

Group	HR (bpm)		PR (ms)		QRS (ms)		QT (ms)		QTc (ms)	
	Before	After	Before	After	Before	After	Before	After	Before	After
***Etomidate***										
**1 ED**_**50**_	161± 6	159±9	87±5.8	89±7.0	36±3.5	33±1.2	253±3	241±21	413±3	392±36
**2 ED**_**50**_	167±16	163±23	85±4.2	88±6.0	32±5.3	32±5.3	245±15	251±16	407±8	410±8
**4 ED**_**50**_	169±13	155±16	82±4.6	84±5.3	36±3.5	33±6.1	244±13	255±8	409±9	409±8
***ET-26 HCl***										
**1 ED**_**50**_	154±16	156±14	88±8.1	87± 6.1	39±4.6	37±4.2	238±10	237±9	380±30	382±31
**2 ED**_**50**_	157±8	175±15	84±2.0	84± 5.3	39±3.1	37±3.1	234±14	236±16	396±20	402±14
**4 ED**_**50**_	167±13	145±18	83±3.1	82± 2.8	33±1.2	39±1.2	227±29	256±25	376±36	396±17

Data are presented as mean ± standard deviation. ED_50_: median effective dose. ECG: electrocardiogram; HR: heart rate; PR: PR interval; QRS: QRS complex; QT: QT interval; QTc: heart rate corrected QT interval. n = 3 for each sub-group.

**Table 5:** Arrhythmia scores and QT-prolongation.

Group	Observed samples	Abnormal samples	QT prolongation	Arrhythmia scores						Incidence of Arrhythmia (%)
				0	1	2	3	4	5	
**Baseline**	18	0/ 18	─	0						0
***Etomidate***										
**1 ED**_**50**_	3	0/ 3	─	0						0
**2 ED**_**50**_	3	0/ 3	─	0						0
**4 ED**_**50**_	3	0/ 3	─	0						0
***ET-26 HCl***										
**1 ED**_**50**_	3	0/ 3	─	0						0
**2 ED**_**50**_	3	0/ 3	─	0						0
**4 ED**_**50**_	3	0/ 3	─	0						0
**Total**	18	0/ 18	─	0						0

Data are presented as mean ± standard deviation. ED_50_: median effective dose. n = 3 for each sub-group.

### *In vitro* studies

#### *In vitro* cardiac function in rat hearts

**Fig 4:**
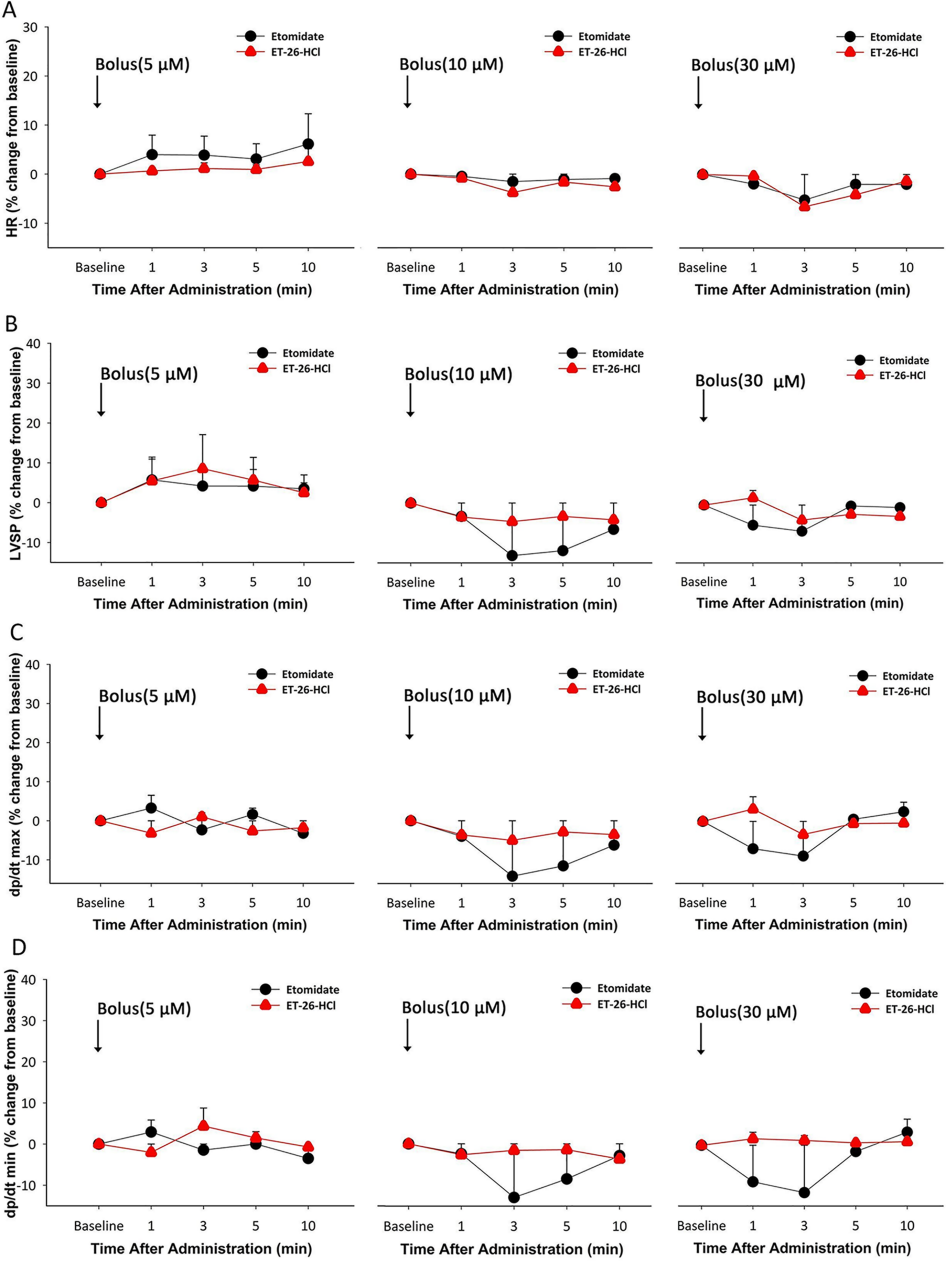
Cardiac parameters change *in vitro* after the administration of ET-26 HCl and etomidate. T = baseline is the time of hypnotic bolus administration. HR: heart rate; LVSP: left ventricular systolic pressure; ± dP/dtmax: maximal rate for left ventricular pressure rising and declining. *(A)* Time-dependent % change in HR from baseline after administering ET-26 HCl and etomidate at the increasing concentrations of 5, 10, and 30 μM. *(B)* Time-dependent % change in LVSP from baseline after administering ET-26 HCl and etomidate at the increasing concentrations of 5, 10, and 30 μM. *(C)* Time-dependent % change in dp/dt_max_ from baseline after administering ET-26 HCl and etomidate at the increasing concentrations of 5, 10, and 30 μM. *(D)* Time-dependent % change in dp/dt_min_ from baseline after administering ET-26 HCl and etomidate at the increasing concentrations of 5, 10, and 30 μM. Data are presented as mean ± standard deviation (n = 6).

In the Langendorff preparation, isolated hearts, from a total of 36 rats with a mean weight of 288 g (range, 264–337g) and age of 8–10 weeks, were used. Before treatment, the basal cardiac parameters were recorded. After treatment using ET-26 HCl or etomidate at 5, 10, or 30 μM, no significant change in *in vitro* cardiac function (HR, LVDP, ±dp/dt_max_) was found between the two groups (Fig 4).

#### *In vitro* electrophysiology study

**Fig 5:**
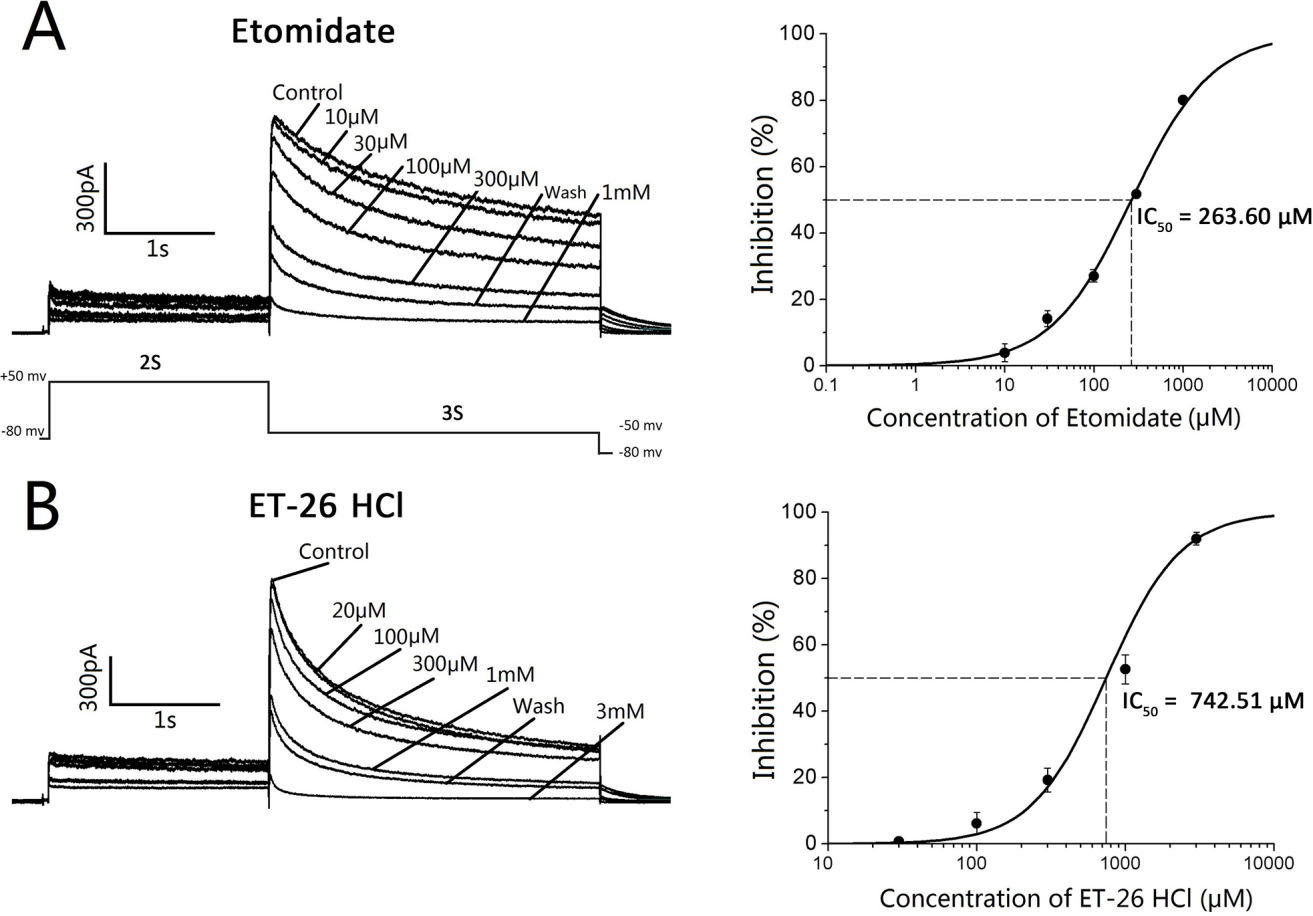
Concentration-response relationships for the inhibition of the hERG by etomidate and its analogue, ET-26 HCl. Whole-cell hERG currents were elicited using a 2 s depolarizing pulse to +50 mV from a holding potential of −80 mV and repolarization to −50 mV for 3 s to measure the tail currents every 30 s in the absence and presence of etomidate (*A*) and ET-26 HCl (*B*). % inhibitions of the hERG tail currents are plotted as increasing concentrations of etomidate and ET-26 HCl. The IC_50_ of etomidate and ET-26 HCl was 263.60 μM and 742.51 μM, respectively. Data are expressed as the mean ± standard deviation. n = 3 for each concentration. IC_50_: the concentration of a compound that inhibits 50% of I_Kr_ activity.

Fig 5 shows the effects of etomidate and its analogue, ET-26 HCl on the hERG currents expressed in HEK-293 cells. Fig 5 demonstrates that etomidate inhibited the tail current of the hERG in a concentration-dependent manner, *i*.*e*., etomidate at concentrations of 10, 30, 100, 300, and 1000 μM reduced the tail current by 3.83 ± 2.36%, 14.13 ± 5.09%, 27.03 ± 4.24%, 51.72 ± 4.12% and 80.06 ± 3.26% (n = 3), respectively. The IC_50_ value was 263.60 μM.

The etomidate analogue, ET-26 HCl, inhibited the hERG current in a similar concentration-dependent manner (Fig 5B). ET-26 HCl at concentrations of 30, 100, 300, 1000, and 3000 μM reduced the tail current by 0.75 ± 0.35%, 6.16 ± 3.2%, 19.27 ± 3.62%, 52.63 ± 4.35%, 91.95 ± 0.24% (n = 3), respectively. The IC_50_ value was 742.51 μM.

## Discussion

The purpose of this study was to evaluate the effect of a single administration of ET-26 HCl on cardiac function when compared with etomidate *in vivo* and *in vitro*. The major finding of the present study was that ET-26 HCl retains the superior myocardial performance of etomidate. To the best of our knowledge, this is the first investigation to define the effect of this etomidate analogue on cardiac performance using echocardiography, combined with assessments of ventricular repolarization and proarrhythmic risk, and an electrophysiology study, to provide new experimental evidence for cardiac safety.

After a single bolus administration of 1×, 2×, 4× ED_50_, the *in vivo* hemodynamics study demonstrated no significant differences in echocardiography and ECG parameters. LV internal diameter, LV volume in diastole and systole and LVEF remained in the normal range.

It is still unclear whether prolongation of the QT interval is associated with the occurrence of drug-induced TdP [26–27], but during the drug development process and cardiac safety evaluation, standard ventricular assays and proarrhythmic assessment of new chemical entities are essential and irreplaceable. During the *in vivo* study, no arrhythmia occurred, and no prolongation of the QT interval was found.

The *in vitro* hemodynamics study was also designed to compare the direct cardiac effects of ET-26 HCl and etomidate by analysing responses at equimolar concentrations. Cardiac function was assessed by measuring LVSP and ±dp/dt_max_. The results demonstrated that etomidate, and its analogue, ET-26 HCl had similar effects on the isolated rat hearts perfused at constant pressure.

The *in vitro* concentrations of etomidate were chosen to correspond with peak plasma concentrations reported *in vivo* in humans. The recommended intravenous dose for induction of anesthesia is approximately 0.3 mg/kg for etomidate, and the peak plasma concentration (C_max_) during induction has been reported to be approximately 3 μM, and researchers have shown that the concentration of etomidate which causes sinus arrest in all hearts is 1000 μM [28–29]. Therefore, we used relatively low concentrations of 5, 10, and 30 μM for the *in vitro* rat hemodynamics study, and 10–3000 μM for the *in vitro* electrophysiology study.

Correspondingly, we designed the *in vitro* I_Kr_ assay and the whole-cell patch-clamp technique to study the effects of ET-26 HCl and etomidate on the hERG channel. The results suggest that etomidate and ET-26 HCl inhibited the tail current of the hERG in a concentration-dependent manner, but the concentrations decreased the channel protein expression at a supra-therapeutic concentration or overdose, with an IC_50_ of 263.60 μM and 742.51 μM, respectively.

While a general relationship between hERG blockade and proarrhythmic risk is generally accepted, an isolated IC_50_ value *in vitro* may be misleading, as multiple factors, such as hypokalaemia, structural heart disease or other cardiac ion channels can mitigate or worsen the risk of QT prolongation [30–31]. Therefore, the IKr IC_50_ measurements must be interpreted in the context of C_max_. If the safety margin (safety margin = IC_50_/C_max_) is 30 or greater, the incidence of TdP is low [32–33]. Although we have not yet characterized the pharmacokinetic behaviour of ET-26 HCl, we can speculate that the safety margin of ET-26 HCl might be as wide as that of etomidate from the current results.

There are also several potential limitations in the current study. Firstly, in order to rule out the possible influence of drug interactions, isolated rat hearts and dogs were selected at random for each sub-group, and each rat or dog was used only for one concentration or dose of one drug, and not for rising concentrations or dosages at intervals of several minutes; therefore, the curves might not reflect a good dose-dependent relationship. Secondly, in the *in vitro* I_kr_ assay, we did not further elucidate cellular mechanisms affecting repolarization and compound activity at other cardiac ion channels. Therefore, future investigations should proceed on the basis of the present study.

In conclusion, using an *in vivo* experiment and a whole organ preparation, we found that ET-26 HCl can retain the superior myocardial performance of etomidate. In addition, the electrophysiology study indicated that ET-26 HCl and etomidate inhibited the hERG at a supra-therapeutic concentration.

## Supporting information

S1 Table The inhibition of the hERG by etomidate at increasing concentrations. (PDF)

S2 Table The inhibition of the hERG by ET-26 HCl at increasing concentrations. (PDF)

S3 Table The % change of HR from baseline in vitro after the administration of ET-26-HCl and etomidate at the increasing concentrations of 5, 10, 30μM. (PDF)

S4 Table The % change of LVDP from baseline in vitro after the administration of ET-26-HCl and etomidate at the increasing concentrations of 5, 10, 30μM. (PDF)

S5 Table The % change of dp/dtmax from baseline in vitro after the administration of ET-26-HCl and etomidate at the increasing concentrations of 5, 10, 30μM. (PDF)

S6 Table The % change of dp/dtmin from baseline in vitro after the administration of ET-26-HCl and etomidate at the increasing concentrations of 5, 10, 30μM. (PDF)

S7 Table TTE parameters after bolus administration of etomidate and ET-26 HCl of 1 ED_50_. (PDF)

S8 Table TTE parameters after bolus administration of etomidate and ET-26 HCl of 2 ED_50_. (PDF)

S9 Table TTE parameters after bolus administration of etomidate and ET-26 HCl of 4 ED_50_. (PDF)

S10 Table ECG parameters after bolus administration of etomidate and ET-26 HCl of 1 ED_50_. (PDF)

S11 Table ECG parameters after bolus administration of etomidate and ET-26 HCl of 2 ED_50_. (PDF)

S12 Table ECG parameters after bolus administration of etomidate and ET-26 HCl of 4 ED_50_. (PDF)
