# Supplementary material for: The etomidate analog ET-26 HCl retains superior myocardial performance: Comparisons with etomidate in vivo and in vitro
Source: PLoS One. 2018 Jan 11;13(1):e0190994. doi: 10.1371/journal.pone.0190994 (PMC5764323; doi:10.1371/journal.pone.0190994)
Supplement: S1 Table — (PDF) [file pone.0190994.s001.pdf]

Concentration	%inhibition				Average	SD
**10 mM**	1.71	6.38	3.40		3.83	2.36
**30 mM**	11.79	21.38	9.72	13.63	14.13	5.09
**100 mM**	23.66	33.23	25.41	25.81	27.03	4.24
**300 mM**	46.20	53.70	55.75	51.21	51.72	4.12
**1 mM**		82.45	81.39	76.34	80.06	3.26
