# Supplementary material for: The etomidate analog ET-26 HCl retains superior myocardial performance: Comparisons with etomidate in vivo and in vitro
Source: PLoS One. 2018 Jan 11;13(1):e0190994. doi: 10.1371/journal.pone.0190994 (PMC5764323; doi:10.1371/journal.pone.0190994)
Supplement: S2 Table — (PDF) [file pone.0190994.s002.pdf]

Concentration	%inhibition			Average	SD
**30 uM**	0.38	0.79	1.08	0.75	0.35
**100 uM**	2.55	7.29	8.65	6.16	3.20
**300 uM**	15.53	22.76	19.53	19.27	3.62
**1 mM**	55.30	47.61	54.97	52.63	4.35
**3 mM**	91.67	92.07	92.12	91.95	0.24
