# Supplementary material for: The etomidate analog ET-26 HCl retains superior myocardial performance: Comparisons with etomidate in vivo and in vitro
Source: PLoS One. 2018 Jan 11;13(1):e0190994. doi: 10.1371/journal.pone.0190994 (PMC5764323; doi:10.1371/journal.pone.0190994)
Supplement: S3 Table — (PDF) [file pone.0190994.s003.pdf]

	**5μM**		**10μM**		**30μM**	
**Time(min)**	etomidate	ET-26 HCl	etomidate	ET-26 HCl	etomidate	ET-26 HCl
**1**	3.98 ± 1.62	0.66 ± 0.27	-0.46 ± 4.31	-0.82 ± 4.39	-1.95 ± 4.23	-0.32 ± 1.70
**3**	3.87 ± 1.58	1.15 ± 0.47	-1.53 ± 1.94	-3.75 ± 1.97	-5.22 ± 5.16	-6.59 ± 3.95
**5**	3.09 ± 1.26	0.96 ± 0.39	-1.11 ± 2.06	-1.62 ± 3.45	-2.03 ± 6.77	-4.14 ± 2.58
**10**	6.16 ± 2.51	2.59 ± 1.06	-0.91 ± 4.39	-2.58 ± 2.30	-2.02 ± 3.90	-1.35 ± 2.18
