# Supplementary material for: The etomidate analog ET-26 HCl retains superior myocardial performance: Comparisons with etomidate in vivo and in vitro
Source: PLoS One. 2018 Jan 11;13(1):e0190994. doi: 10.1371/journal.pone.0190994 (PMC5764323; doi:10.1371/journal.pone.0190994)
Supplement: S4 Table — (PDF) [file pone.0190994.s004.pdf]

	5μM		10μM		30μM	
**Time(min)**	etomidate	ET-26 HCl	etomidate	ET-26 HCl	etomidate	ET-26 HCl
**1**	5.72 ± 2.33	5.47 ± 2.23	-3.35 ± 8.94	-3.50 ± 4.50	-5.21 ± 6.55	1.90 ± 1.39
**3**	4.18 ± 1.71	8.54 ± 3.49	-13.17 ± 9.51	-4.64 ± 4.44	-6.77 ± 7.66	-3.89 ± 3.10
**5**	4.16 ± 1.70	5.68 ± 2.32	-11.93 ± 8.64	-3.34 ± 4.43	-0.22 ± 2.94	-2.39 ± 4.98
**10**	3.49 ± 1.42	2.49 ± 1.02	-6.61 ± 2.93	-4.19 ± 3.04	-0.63 ± 2.23	-2.93 ± 1.36
