# Supplementary material for: The etomidate analog ET-26 HCl retains superior myocardial performance: Comparisons with etomidate in vivo and in vitro
Source: PLoS One. 2018 Jan 11;13(1):e0190994. doi: 10.1371/journal.pone.0190994 (PMC5764323; doi:10.1371/journal.pone.0190994)
Supplement: S5 Table — (PDF) [file pone.0190994.s005.pdf]

	5μM		10μM		30μM	
**Time(min)**	etomidate	ET-26 HCl	etomidate	ET-26 HCl	etomidate	ET-26 HCl
**1**	3.26 ± 5.83	-3.16 ± 6.09	-3.99 ± 13.49	-3.61 ± 5.87	-7.06 ± 9.70	3.18 ± 1.48
**3**	-2.34 ± 4.28	1.03 ± 9.49	-14.14 ± 12.13	-5.00 ± 5.22	8.94 ± 13.15	-3.38 ± 4.20
**5**	1.62 ± 5.48	-2.60 ± 6.14	-11.52 ± 10.57	-2.85 ± 5.65	0.54 ± 6.05	-0.57 ± 5.60
**10**	-3.21 ± 7.61	-1.79 ± 3.10	-6.23 ± 6.20	-3.53 ± 3.60	2.49 ± 1.37	-0.44 ± 5.60
