# Supplementary material for: The etomidate analog ET-26 HCl retains superior myocardial performance: Comparisons with etomidate in vivo and in vitro
Source: PLoS One. 2018 Jan 11;13(1):e0190994. doi: 10.1371/journal.pone.0190994 (PMC5764323; doi:10.1371/journal.pone.0190994)
Supplement: S6 Table — (PDF) [file pone.0190994.s006.pdf]

Time(min)	5μM		10μM		30μM	
	etomidate	ET-26 HCl	etomidate	ET-26 HCl	etomidate	ET-26 HCl
**1**	2.93 ± 6.42	-2.02 ± 5.61	-2.40 ± 9.57	-2.63 ± 3.84	-9.03 ± 12.04	1.61 ± 3.50
**3**	-1.46 ± 5.80	4.39 ± 7.91	-12.94 ± 14.85	-1.57 ± 2.65	-11.70 ± 17.94	1.20 ± 5.47
**5**	0.01 ± 6.69	1.50 ± 5.76	-8.47 ± 10.75	-1.41 ± 5.23	-1.55 ± 13.36	0.59 ± 5.84
**10**	-3.49 ± 10.04	-0.71 ± 3.26	-2.86 ± 4.80	-3.66 ± 2.84	3.24 ± 2.18	0.90 ± 1.77
