# Supplementary material for: The etomidate analog ET-26 HCl retains superior myocardial performance: Comparisons with etomidate in vivo and in vitro
Source: PLoS One. 2018 Jan 11;13(1):e0190994. doi: 10.1371/journal.pone.0190994 (PMC5764323; doi:10.1371/journal.pone.0190994)
Supplement: S7 Table — (PDF) [file pone.0190994.s007.pdf]

| | Group | LVIDd (mm) | EDV (ml) | LVIDs (mm) | ESV (ml) | EF (%) |
|---|---|---|---|---|---|---|
| | ***etomidate*** | | | | | |
| **Animal Number** | **NO.16** | 31.94 | 32.848 | 25.36 | 16.398 | 50.02 |
| | **NO.17** | 30.56 | 28.802 | 16.76 | 4.764 | 83.44 |
| | **NO.21** | 39.2 | 60.244 | 30.2 | 27.664 | 54.08 |
| | ***ET-26 HCl*** | | | | | |
| **Animal Number** | **NO.33** | 32.16 | 33.344 | 20.76 | 9.042 | 72.92 |
| | **NO.37** | 32.88 | 35.776 | 24.68 | 15.23 | 57.62 |
| | **NO.28** | 38.06 | 55.122 | 27.86 | 21.71 | 60.68 |
