# Supplementary material for: The etomidate analog ET-26 HCl retains superior myocardial performance: Comparisons with etomidate in vivo and in vitro
Source: PLoS One. 2018 Jan 11;13(1):e0190994. doi: 10.1371/journal.pone.0190994 (PMC5764323; doi:10.1371/journal.pone.0190994)
Supplement: S8 Table — (PDF) [file pone.0190994.s008.pdf]

	Group	LVIDd (mm)	EDV (ml)	LVIDs (mm)	ESV (ml)	EF (%)
	etomidate					
**Animal Number**	**NO.19**	36.06	46.712	27.64	21.06	54.74
	**NO.20**	31.26	30.672	22.46	11.348	62.76
	**NO.23**	31.7	31.862	19.58	7.516	76.36
	ET-26 HCl					
**Animal Number**	**NO.3**	35.48	44.968	23.46	13.126	71.02
	**NO.8**	30.9	31.61	19.94	8.012	73.04
	**NO.9**	31	30.362	19.54	7.952	74.82
