# Supplementary material for: The etomidate analog ET-26 HCl retains superior myocardial performance: Comparisons with etomidate in vivo and in vitro
Source: PLoS One. 2018 Jan 11;13(1):e0190994. doi: 10.1371/journal.pone.0190994 (PMC5764323; doi:10.1371/journal.pone.0190994)
Supplement: S9 Table — (PDF) [file pone.0190994.s009.pdf]

	Group	LVIDd (mm)	EDV (ml)	LVIDs (mm)	ESV (ml)	EF (%)
	etomidate					
**Animal Number**	**NO.32**	32.16	33.344	20.76	9.042	72.92
	**NO.34**	33.06	36.242	26	17.554	51.42
	**NO.36**	34.16	40.386	23.88	13.842	66.02
	ET-26 HCl					
**Animal Number**	**NO.13**	39.66	62.794	29.94	27.038	56.88
	**NO.14**	33.12	36.356	25.5	16.59	54.32
	**NO.39**	28.68	23.57	20.1	8.192	65.38
