# Supplementary material for: The etomidate analog ET-26 HCl retains superior myocardial performance: Comparisons with etomidate in vivo and in vitro
Source: PLoS One. 2018 Jan 11;13(1):e0190994. doi: 10.1371/journal.pone.0190994 (PMC5764323; doi:10.1371/journal.pone.0190994)
Supplement: S10 Table — (PDF) [file pone.0190994.s010.pdf]

	Group	HR (bpm)	PR (ms)	QRS (ms)	QT (ms)	QTc (ms)
	etomidate					
**Animal Number**	**NO.16**	156	82	34	218	351
	**NO.17**	152	88	32	258	410
	**NO.21**	169	96	34	248	415
	ET-26 HCl					
**Animal Number**	**NO.33**	147	80	42	238	372
	**NO.37**	172	92	36	246	416
	**NO.28**	148	88	34	228	357
