# Supplementary material for: The etomidate analog ET-26 HCl retains superior myocardial performance: Comparisons with etomidate in vivo and in vitro
Source: PLoS One. 2018 Jan 11;13(1):e0190994. doi: 10.1371/journal.pone.0190994 (PMC5764323; doi:10.1371/journal.pone.0190994)
Supplement: S11 Table — (PDF) [file pone.0190994.s011.pdf]

	Group	HR (bpm)	PR (ms)	QRS (ms)	QT (ms)	QTc (ms)
	etomidate					
**Animal**	**NO.19**	174	88	38	246	418
**Number**	**NO.20**	136	94	28	268	403
	**NO.23**	178	82	30	238	409
	ET-26 HCl					
**Animal**	**NO.3**	179	90	40	224	386
**Number**	**NO.8**	188	80	34	230	407
	**NO.9**	159	82	36	254	413
