# Supplementary material for: The etomidate analog ET-26 HCl retains superior myocardial performance: Comparisons with etomidate in vivo and in vitro
Source: PLoS One. 2018 Jan 11;13(1):e0190994. doi: 10.1371/journal.pone.0190994 (PMC5764323; doi:10.1371/journal.pone.0190994)
Supplement: S12 Table — (PDF) [file pone.0190994.s012.pdf]

	Group	HR (bpm)	PR (ms)	QRS (ms)	QT (ms)	QTc (ms)
	etomidate					
**Animal Number**	**NO.32**	141	82	28	262	401
	**NO.34**	167	88	32	248	413
	**NO.36**	153	90	40	258	411
	ET-26 HCl					
**Animal Number**	**NO.13**	143	86	40	264	407
	**NO.14**	164	80	38	228	376
	**NO.39**	129	84	38	276	404
